# Energy-Efficient Message Bundling with Delay and Synchronization Constraints in Wireless Sensor Networks

**DOI:** 10.3390/s22145276

**Published:** 2022-07-14

**Authors:** Sihao Li, Kyeong Soo Kim, Linlin Zhang, Xintao Huan, Jeremy Smith

**Affiliations:** 1 School of Advanced Technology, Xi’an Jiaotong-Liverpool University (XJTLU), Suzhou 215123, China; sihao.li19@student.xjtlu.edu.cn (S.L.); linlin.zhang18@alumni.xjtlu.edu.cn (L.Z.); 2 Department of Electrical Engineering and Electronics, University of Liverpool, Liverpool L69 3BX, UKj.s.smith@liverpool.ac.uk (J.S.); 3 School of Cyberspace Science and Technology, Beijing Institute of Technology, Beijing 100081, China; xintao.huan@bit.edu.cn

**Keywords:** message bundling, energy efficiency, wireless sensor networks (WSNs), end-to-end delay, time synchronization accuracy

## Abstract

In a wireless sensor network (WSN), reducing the energy consumption of battery-powered sensor nodes is key to extending their operating duration before battery replacement is required. Message bundling can save on the energy consumption of sensor nodes by reducing the number of message transmissions. However, bundling a large number of messages could increase not only the end-to-end delays and message transmission intervals, but also the packet error rate (PER). End-to-end delays are critical in delay-sensitive applications, such as factory monitoring and disaster prevention. Message transmission intervals affect time synchronization accuracy when bundling includes synchronization messages, while an increased PER results in more message retransmissions and, thereby, consumes more energy. To address these issues, this paper proposes an optimal message bundling scheme based on an objective function for the total energy consumption of a WSN, which also takes into account the effects of packet retransmissions and, thereby, strikes the optimal balance between the number of bundled messages and the number of retransmissions given a link quality. The proposed optimal bundling is formulated as an integer nonlinear programming problem and solved using a self-adaptive global-best harmony search (SGHS) algorithm. The experimental results, based on the Cooja emulator of Contiki-NG, demonstrate that the proposed optimal bundling scheme saves up to 51.8% and 8.8% of the total energy consumption with respect to the baseline of no bundling and the state-of-the-art integer linear programming model, respectively.

## 1. Introduction

A typical wireless sensor network (WSN) consists of a head node with abundant computing and power resources and a large number of resource-constrained, battery-powered sensor nodes [1,2]. As the sensor nodes’ energy is strictly limited by the equipped batteries, minimizing their energy consumption is critical to the operation of the entire WSN; for instance, the lifetime of a WSN could be extended by up to 52% through the use of energy-efficient transmission algorithms and protocols [3].

The number of message transmissions can be reduced by bundling several messages together and transmitting them in a common data frame or packet [4,5]. As data transmission modules consume the most energy [6,7], message bundling is considered an efficient technique for reducing the energy consumption of a sensor node [8,9]. However, message bundling not only increases the end-to-end (E2E) delay, but also reduces the synchronization accuracy [10,11,12,13,14]. Various optimal message bundling schemes that investigate the relationship between message bundling and the E2E delay in the reduction of energy consumption have been proposed. However, in most of the work, the message bundling is considered together with, or as part of, other protocols and processes, such as routing [8,9], scheduling [15], and query processing [16]. Few of them focus on message bundling and its effect on energy consumption, both directly and independently.

In [17], for the first time, we took into account time synchronization accuracy and E2E delays in optimal message bundling, where the energy consumption is indirectly minimized by maximizing the total number of bundled messages in a WSN through computationally feasible integer linear programming (ILP). Unlike most of the existing work, in this study, we purely focus on the optimal message bundling, independently from other protocols, such as routing and scheduling. Though the indirect minimization of the energy consumption dramatically simplifies the formulation of the optimal bundling, it cannot capture the negative effect of message bundling on the energy consumption, i.e., a higher probability of retransmissions due to the increased packet error rate (PER) [6]. In this paper, we formulate the optimal bundling problem with a new objective function, thus directly modeling the total energy consumption of a WSN, to address the issue of the optimal message bundling scheme proposed in [17], which takes into account both the positive and negative effects of message bundling on the energy consumption and, thereby, strikes the optimal balance between the number of bundled messages and the number of retransmissions given the link quality. We solve the resulting integer nonlinear programming (INLP) problem using a self-adaptive global-best harmony search (SGHS) [18,19].

As for the verification of the energy efficiency of message bundling schemes, the direct measurement of energy consumption through experiments with a real testbed would not be feasible because hardware-based energy measurement is too difficult to implement due to the significant number of modifications required for existing hardware [20,21] and the need for a dedicated circuit for the measurement [22]. To make matters worse, the interconnection with measurement devices—such as oscilloscopes and digital multimeters—would make it challenging to guarantee that the targets are working under normal conditions. Emulation can provide an alternative solution in this regard; unlike simulation, emulation is based on software implementations (i.e., firmware in our case) that can run on original devices (i.e., WSN motes) without any modification, so experimental results from emulation are more convincing and credible than those from simulation. In addition, measuring energy consumption is more straightforward with emulation than with actual hardware. Therefore, in this paper, considering the diversity of WSN devices and the ease of measuring energy consumption, we verify the energy efficiency of the proposed bundling scheme through realistic experiments based on the Cooja emulator of Contiki-NG [23].

The major contribution of our work in this paper is two-fold: First, we propose a new energy-efficient optimal message bundling scheme where we formulate the optimal message bundling based on an objective function modeling the total power consumption of the whole WSN. The proposed scheme, for the first time, enables us to directly minimize the energy consumption under the joint constraints of synchronization accuracy and E2E delay and to investigate the negative effects of message bundling—i.e., increased PER and the number of retransmissions—on the energy consumption. We also apply the advanced SGHS algorithm in order to solve the resulting INLP problem.

Second, we carry out a comparative analysis of the optimal bundling schemes, where we compare the energy consumption of the three different schemes—i.e., the proposed scheme, the ILP model of [17], and the baseline of no bundling—based on the Contiki-NG and its Cooja emulator with eight different network topologies. We also calculate the power consumption of the three schemes based on a numerical analysis for the verification of the emulation results.

The rest of the paper is organized as follows: The objective function and constraints of the optimal bundling problem are described in Section 2. The emulation process is discussed in Section 3. The experimental results and related discussions are presented in Section 4. Section 5 concludes our work in this paper.

## 2. Energy-Efficient Optimal Message Bundling

In formulating the energy-efficient optimal message bundling problem, we consider a WSN consisting of one head node and *N* sensor nodes. Each sensor node periodically generates a measurement message with length $L_{M}$ during each measurement interval (MI). The length $L^{i}$ of a packet bundling $\Gamma^{i}$ messages at node *i* (i∈[0,1,…,N−1]) is given by

(1) $$L^{i}=L_{H}+\Gamma^{i}\;\cdot \;L_{M},$$

where $L_{H}$ is the length of a packet header and, if it exists, a packet footer.

**Figure 1:**
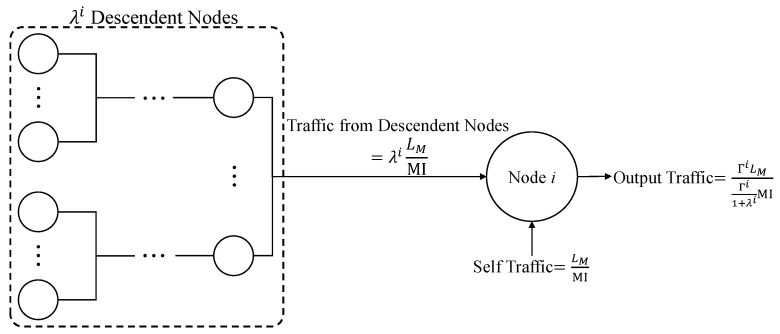
Conservation of traffic flows at a non-leaf node.

If node *i* is not a leaf node and has $\lambda^{i}$ descendant nodes, as shown in Figure 1, the period for generating a packet bundling $\Gamma^{i}$ messages should be $\frac{\Gamma^{i}}{1+\lambda^{i}}\mathrm{MI}$ from the conservation of traffic flows [17]. Here, we assume that, at each non-leaf node, the messages of incoming packets are unbundled first and bundled again with the measurement messages generated at the node before being transmitted via outgoing packets.

### 2.1. Objective Function

Equation (1) suggests that the energy-saving in message bundling mainly comes from the saved energy for $\Gamma^{i}-1$ headers. To quantify the energy consumption in message bundling, we use the amount of energy required for the transmission of one *information bit* based on the PER, i.e., the ratio of the number of unacknowledged packets to the total number of transferred packets, which is defined as follows [6]:

(2) $$E_{b}=\frac{\left(L_{H}+\Gamma^{i}\;\cdot \;L_{M}\right)}{\Gamma^{i}\;\cdot \;L_{M}\;\cdot \;\left(1-\mathrm{PER}\right)}\;\cdot \;E_{TX},$$

where $E_{TX}$ is the energy consumption for transmitting one bit of data at a given output power level $P_{TX}$. $E_{TX}$ can be obtained via $\frac{P_{TX}}{R_{TX}}$, where $R_{TX}$, the transmission rate specified in the IEEE 802.15.4 standard, is 250 kbps. In [6], the PER is empirically modeled as a function of the signal-to-noise ratio (SNR), i.e.,

(3) $$\mathrm{PER}=\alpha \;\cdot \;e^{\beta \;\cdot \;\mathrm{SNR}}\;\cdot \;\Gamma^{i}\;\cdot \;L_{M},$$

where α = 0.0128 and β = −0.15. From Equations (2) and (3), we obtain:

(4) $$E_{b}=\frac{E_{TX}\;\cdot \;\left(L_{H}+\Gamma^{i}\;\cdot \;L_{M}\right)}{\Gamma^{i}\;\cdot \;L_{M}\;\cdot \;\left(1-0.0128\;\cdot \;e^{-0.15\mathrm{SNR}}\;\cdot \;\Gamma^{i}\;\cdot \;L_{M}\right)}.$$

The energy consumption for transmitting a packet bundling $\Gamma^{i}$ messages at node *i* is given by

(5) $$\begin{array}{cc} E_{TX}^{i} & =L^{i}\;\cdot \;E_{b}\\ \; & =\frac{E_{TX}\;\cdot \;{\left(L_{H}+\Gamma^{i}\;\cdot \;L_{M}\right)}^{2}}{\Gamma^{i}\;\cdot \;L_{M}\;\cdot \;\left(1-0.0128\;\cdot \;e^{-0.15\mathrm{SNR}}\;\cdot \;\Gamma^{i}\;\cdot \;L_{M}\right)}. \end{array}$$

Given the bundling number $\Gamma^{i}$ and the number of descendant nodes $\lambda^{i}$, the bundled packet transmission interval at node *i* is given by $\frac{\Gamma^{i}}{1+\lambda^{i}}\mathrm{MI}$, during which the amount of energy $E_{TX}^{i}$ is consumed. Therefore, the average power consumption for packet transmission at node *i* is given by

(6) $$\begin{array}{cc} P_{TX}^{i}\left(\Gamma^{i}\right) & =\frac{E_{TX}^{i}}{\frac{\Gamma^{i}}{1+\lambda^{i}}\;\cdot \;\mathrm{MI}}\\ \; & =\frac{\left(1+\lambda^{i}\right)\;\cdot \;E_{TX}\;\cdot \;{\left(L_{H}+L_{M}\;\cdot \;\Gamma^{i}\right)}^{2}}{{\left(\Gamma^{i}\right)}^{2}\;\cdot \;\mathrm{MI}\;\cdot \;L_{M}\;\cdot \;\left(1-0.0128\;\cdot \;e^{-0.15\mathrm{SNR}}\;\cdot \;\Gamma^{i}\;\cdot \;L_{M}\right)}. \end{array}$$

Note that the transmission of a bundled packet at a node causes the energy consumption for the reception of the corresponding packet at its destination node(s); there could be multiple destinations in the case of multicast and broadcast. However, the energy consumption for receiving a packet could be different from that for transmitting the same packet because the transceiver module in a typical WSN mote is designed with asymmetric current consumption. Considering this difference in current consumption for transmission and reception, we can obtain the average power consumption for packet reception at node *i* under the assumption of a common supply voltage for all WSN nodes as follows:

(7) $$P_{RX}^{i}=\sigma \sum_{j\in C^{i}}P_{TX}^{j}\left(\Gamma^{j}\right),$$

where σ is a ratio between current consumption for reception and transmission (i.e., $\frac{I_{RX}}{I_{TX}}$) and $C^{i}$ is a set of indexes of node *i*’s child nodes. Therefore, the average power consumption at node *i* is given by

(8) $$P^{i}=P_{TX}^{i}+P_{RX}^{i}.$$

As the average power consumption is stable and constant under periodic message generation at all nodes, we can minimize the energy consumption by minimizing the average power consumption. Therefore, we define the objective function of the energy-efficient optimal bundling problem as the total power consumption of the network, i.e.,

(9) $$P^{total}\left(\Gamma \right)=\sum_{i=0}^{N-1}P^{i},$$

where $\Gamma ≜\left[\Gamma^{0},\;\ldots ,\;\Gamma^{N-1}\right]$. Note that, if there are only *unicast transmissions* in the WSN, Equation (9) can be simplified as follows:

(10) $$\begin{array}{cc} P^{total}\left(\Gamma \right) & =\sum_{i=0}^{N-1}P_{TX}^{i}\left(\Gamma^{i}\right)+\sigma \sum_{i=0}^{N-1}\left(\sum_{j\in C^{i}}P_{TX}^{j}\left(\Gamma^{j}\right)\right)\\ \; & =\left(1+\sigma \right)\sum_{i=0}^{N-1}P_{TX}^{i}\left(\Gamma^{i}\right). \end{array}$$

### 2.2. Bundling Constraints

Because 0 ≤ PER ≤ 1, the bundling number $\Gamma^{i}$ in Equation (3) with the values of α and β should satisfy the following condition:

(11) $$\begin{array}{cc} 0 & \leq 0.0128\;\cdot \;e^{-0.15\mathrm{SNR}}\;\cdot \;\Gamma^{i}\;\cdot \;L_{M}\leq 1\\ 0 & \leq \Gamma^{i}\leq \frac{e^{0.15\mathrm{SNR}}}{0.0128\;\cdot \;L_{M}}. \end{array}$$

In practical implementations, the maximum bundling number is also limited by the maximum payload length of the underlying protocols (e.g., 110 bytes in the IEEE 802.15.4 standard [24]). Therefore, we constrain the bundling number $\Gamma_{i}$ as follows:

(12) $$\chi^{min}\leq \Gamma^{i}\leq \min\left(\chi^{max},\frac{e^{0.15\mathrm{SNR}}}{0.0128\;\cdot \;L_{M}}\right),$$

where $\chi^{min}$ is a user-defined minimum bundling number, which is typically 1, and $\chi^{max}$ is the maximum bundling number determined by the ultimate payload length $L_{P,max}$ and the message length $L_{M}$, i.e.,

(13) $$\chi^{max}=\left\lfloor \frac{L_{P,max}}{L_{M}}\right\rfloor .$$

### 2.3. Delay Constraints

While minimizing the energy consumption, we also need to meet the E2E delay and time synchronization accuracy requirements for time-sensitive applications and the proper operation of WSNs. In [17], the joint constraints of E2E delay and time synchronization accuracy are formulated as follows:

(14) $$D_{e2e}^{i}\leq \min\left(D_{e2e}^{max},D_{e2e}^{SA}\right),$$

where $D_{e2e}^{i}$ is the E2E delay of node *i* given by

(15) $$D_{e2e}^{i}=\sum_{l=0}^{L-1}\frac{\Gamma^{i}}{1+\lambda^{i}}\;\cdot \;\mathrm{MI},$$

$D_{e2e}^{max}$ is a user-defined E2E delay requirement, and $D_{e2e}^{SA}$ is another E2E delay requirement translated from the minimum time synchronization interval $SA^{min}$ through the function τ(·), i.e.,

(16) $$D_{e2e}^{SA}=\tau \left(SA^{min}\right).$$

For detailed discussions on the joint constraints of E2E delay and time synchronization accuracy, readers are referred to [17].

### 2.4. Integer Nonlinear Programming Model

With the objective function and the constraints in Equations (10), (12) and (14), the energy-efficient optimal bundling can be formulated as the following INLP problem:

(17) $$\begin{array}{cc} \; & \underset{\Gamma }{\mathrm{minimize}}\;\;P^{total}\left(\Gamma \right)\\ \; & \mathrm{subject}\;\mathrm{to}\\ \; & \;\;\;\;\;\begin{array}{ccc} \; & \chi^{min}\leq \Gamma^{i}\leq \min\left(\chi^{max},\frac{e^{0.15\mathrm{SNR}}}{0.0128\;\cdot \;L_{M}}\right),\; & \forall i\in [0,\;\ldots ,\;N-1],\\ \; & D_{e2e}^{i}\leq \min\left(D_{e2e}^{max},D_{e2e}^{SA}\right),\; & \forall i\in [0,\;\ldots ,\;N-1]. \end{array} \end{array}$$

Note that, thanks to the objective function $P^{total}\left(\Gamma \right)$ modeling the total power consumption of the network, we can now directly minimize the energy consumption in the optimal bundling formulated in Equation (17). The tradeoff of this direct minimization of the energy consumption is that, unlike the formulation in [17], it results in the INLP problem. The details of the approach to solving the INLP problem will be discussed in Section 4.1.

## 3. Cooja-Based Emulation

The rapid development of emulation technologies with ever-increasing computing power enables us to develop, run, test, and debug unmodified embedded software on our PC from chips to independent systems and complex multi-node networks. Unlike traditional network simulators, such as ns-2 [25] and OMNeT++ [26], an emulator can run firmware developed for physical devices (e.g., WSN motes), which eliminates the need for creating and maintaining separate simulation models, thereby resulting in credible experimental results [27,28]. Contiki-NG [23] and TinyOS [29,30] are two popular operating systems (OSs) for WSN and Internet of Things (IoT) devices that provide emulators for the devices that they support.

Cooja is the emulator from Contiki-NG, the OS for next-generation IoT devices, which can provide a development and testing environment for WSN/IoT devices with a powerful graphical user interface (GUI) and network simulation capability [23]. Cooja compiles Contiki-NG to a native platform as a shared library and loads the library via Java Native Interfaces (JNIs) to provide the loaded firmware with the same running environment as the actual devices (i.e., the emulation target). Experiments based on the Cooja emulator can enable more realistic investigations of the effect of optimal bundling on energy consumption than those based on network simulators. TinySim is an emulator from TinyOS, another competitive OS designed explicitly for power-constrained sensor nodes [29,30], but it is not comparable to Cooja in terms of features and functionalities, which is why even TinyOS developers use Cooja to run and test firmware developed with TinyOS. Table 1 provides a short comparison between Contiki-NG and TinyOS.

Though Contiki-NG and TinyOS provide similar functionalities and protocol support, Contiki-NG has more active developer communities and abundant application scenarios from which one can expect more comprehensive technical support. Therefore, for the evaluation of the proposed optimal bundling algorithm, we chose Contiki-NG and Cooja.

One of the major strengths of Cooja is its ability to estimate the energy consumption of WSN motes without the actual deployment of a WSN; there are two options in this regard, i.e., Energest [21], a Contiki-NG time recorder for energy estimation providing linear analysis mechanisms, and PowerTracker [34], a Cooja plugin for energy monitoring. Of the two, because PowerTracker is tightly integrated into the Cooja emulator with the full support of a GUI, the energy consumption calculation is mainly based on the output from PowerTracker, while the data from Energest are used for cross-validation. As for the emulation target, the Z1 [27,35] platform is used to obtain reliable results from the energy consumption calculation based on the up-to-date and detailed data from its datasheet. Table 2 summarizes the software and hardware components for the experiments based on the Cooja emulator.

### Protocol Stack

**Figure 2:**
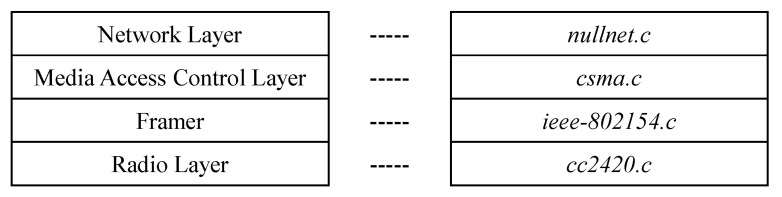
Contiki-NG network protocol stack.

Figure 2 shows the network protocol stack of the WSN motes based on NullNet, the minimal network layer of Contiki-NG [36,37].

We choose NullNet to minimize the effect of the complicated network layer protocols, including routing and encryption, in investigating the impact of message bundling on the energy consumption at sensor nodes, which ensures that most of the energy is used for bundled message transmissions. Likewise, in this paper, we also choose the simple carrier sense multiple access (CSMA) medium access control (MAC) protocol to isolate the effect of message bundling in our investigation of the energy consumption.

## 4. Performance Evaluation

**Figure 3:**
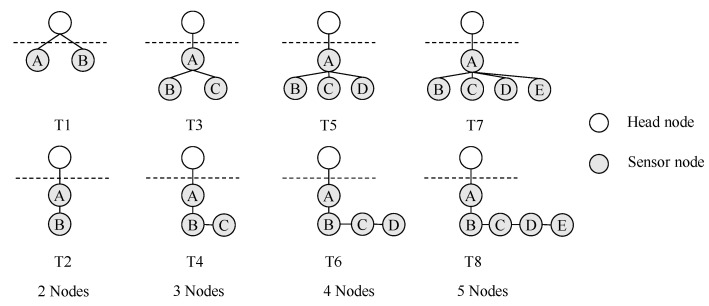
WSN topologies for performance evaluation.

To evaluate the performance of the proposed energy-efficient optimal bundling algorithm, in comparison with the baseline case without message bundling and the ILP model of [17], we consider the eight topologies shown in Figure 3.

We also assume the parameter values summarized in Table 3 for the WSN and optimal bundling unless explicitly stated otherwise.

### 4.1. Optimal Bundling Numbers

Various evolutionary algorithms (EAs)—i.e., population-based optimization algorithms inspired by biological evolution—have been widely used for research on energy conservation in WSNs; for example, a genetic algorithm (GA) was applied to an adaptive clustering protocol to achieve optimal performance in terms of WSN lifetime in [39], particle swarm optimization (PSO) was used for a novel coverage control to reduce the energy consumption of WSN motes in [40], and a social spider optimization (SSO) algorithm was proposed for a clustering a sensor network in [41]. To solve the INLP model formulated in Equation (17), we use SGHS, an improved version of the harmony search (HS) algorithm. HS is a novel intelligent optimization algorithm inspired by the process of improvisation of music performed by an orchestra and has the following advantages over the popular GAs [42,43]:

- HS algorithms are simple, easy to implement, and based on decimal encoding, while GAs mostly use binary encoding and, thereby, suffer from the Hamming Cliff problem [44].
- HS algorithms make the most of both local and global information and can store individual optimal solutions.
- It is convenient to mix HS algorithms with other optimization methods to construct better algorithms.

HS algorithms, however, cannot achieve optimal performance consistently. Therefore, SGHS—which is based on two other variants of HS, i.e., improved HS (IHS) and global-best HS (GHS)—employs a new improvisation scheme and an adaptive parameter tuning method [18,19]. The parameter values of SGHS for optimal bundling are summarized in Table 4.

Based on these parameter values, we obtain the optimal bundling numbers in Table 5 for the eight topologies shown in Figure 3.

The results in Table 5 show that the bundling numbers from the proposed INLP and those from [17] are different for the 1-ary tree topologies (also called *parking lot* topologies [45]) of T2, T4, T6, and T8, but are identical for the other tree topologies of T1, T3, T5, and T7. As the objective function in the INLP (i.e., Equations (6) and (10)) can take into account both the positive and negative effects of message bundling on energy consumption, the INLP results in a bundling of numbers that are more evenly spread than those with the ILP as the height of the tree increases. The energy consumption with the different bundling numbers for the 1-ary trees is analyzed in Section 4.2.

### 4.2. Energy Consumption

To investigate the effect of message bundling in a realistic environment with information on the different operation modes of specific hardware platforms, we measured the energy consumption of WSN nodes based on experiments on a directed graph radio medium (DGRM) [46,47] using a Cooja emulation, where the Z1 mote [35] was selected as a target device for all of the nodes. The current consumption at different states of the Z1 mote is summarized in Table 6.

During the emulation experiments, we did not consider other power-saving mechanisms, such as sleep/wake-up scheduling [48], but used only the two active states of “Radio RX” and “Radio TX” to focus on the effect of message bundling on energy consumption. In this case, the energy consumption of the node can be calculated from the two different operating modes of transmission (TX) and reception (RX) as follows:

(18) $$E^{i}=V\;\cdot \;I_{TX}\;\cdot \;T_{TX}+V\;\cdot \;I_{RX}\;\cdot \;T_{RX},$$

where *V* is the common supply voltage (i.e., 3 V), and $I_{OP}$ and $T_{OP}$ (OP∈[TX,RX]) are the current and the period of each operation mode, respectively. The total energy consumption is given by

(19) $$E^{total}=\sum_{i=0}^{N-1}\left(V\;\cdot \;I_{TX}\;\cdot \;T_{TX}+V\;\cdot \;I_{RX}\;\cdot \;T_{RX}\right).$$

Based on Equation (19), Table 6, and the experimental log files from PowerTracker, we could calculate the total energy consumption for each topology under different message bundling schemes.

**Figure 4:**
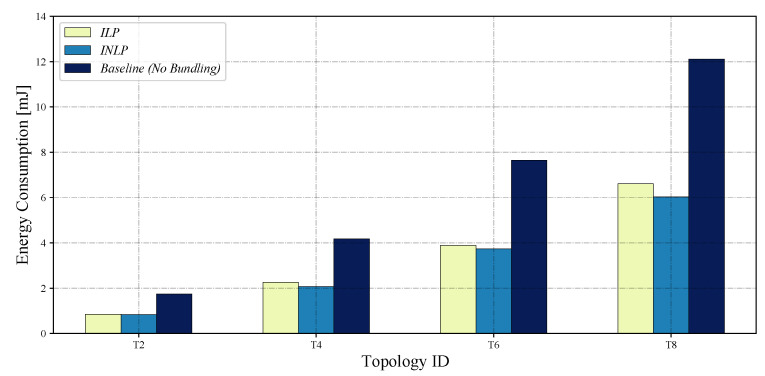
Total energy consumption based on the Cooja emulation.

The emulation experiments ran for 1 h in emulation time with the optimal bundling numbers from INLP and ILP summarized in Table 5, whose total energy consumption results for T2, T4, T6, and T8 are shown in Figure 4; the total power consumption for the case of no message bundling is also shown as a baseline.

**Figure 5:**
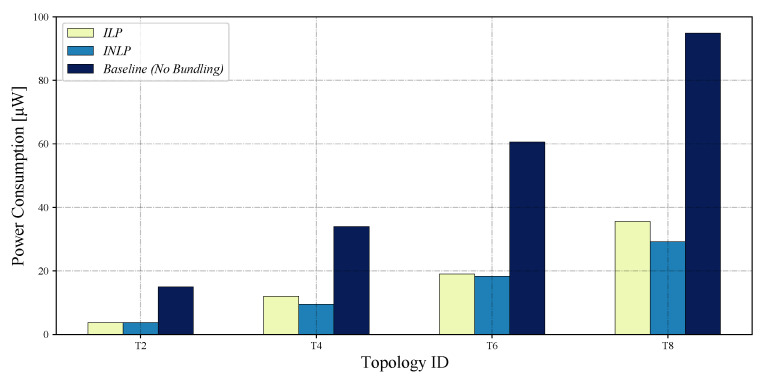
Total power consumption based on Equation (10).

The emulation results confirm that, while both schemes could save a significant amount of energy compared to the baseline, the proposed INLP outperformed the ILP overall. Comparing the emulation results with the mathematical ones shown in Figure 5, which are for total power consumption and based on Equation (10), we can observe that both results show similar trends, indicating that Equation (10) models the total power consumption well under a realistic environment.

To quantify the energy savings of a message bundling scheme with respect to the baseline of no bundling, a performance measure $\eta_{base}$ was defined as follows:

(20) $$\eta_{base}≜\frac{E_{base}^{total}-E_{scheme}^{total}}{E_{base}^{total}}=\frac{E_{base}^{total}-E_{scheme}^{total}}{E_{base}^{total}}.$$

To further analyze the relative energy savings of INLP in comparison to ILP, we also defined another performance measure $\eta_{ILP}$ as follows:

(21) $$\eta_{ILP}≜\frac{E_{ILP}^{total}-E_{INLP}^{total}}{E_{ILP}^{total}}=\frac{E_{ILP}^{total}-E_{INLP}^{total}}{E_{ILP}^{total}}.$$

Table 7 summarizes the total energy consumption saved by the proposed optimal message bundling scheme in comparison to the baseline (no bundling) and the ILP model of [17].

The results clearly show that message bundling could significantly save the total energy consumption for the 1-ary tree topologies. Of the two bundling schemes, the total energy consumption for the proposed optimal bundling scheme based on INLP is smaller than that for the state-of-the-art one based on ILP in all the cases considered. Overall, the proposed bundling scheme can save up to 51.8% and 8.8% of the total energy consumption with respect to the baseline of no bundling and the bundling based on ILP, respectively, as shown in Table 7. As discussed before, this is because the proposed optimal bundling scheme can take into account both the positive and negative effects of message bundling on the energy consumption during the optimization thanks to the objective function given in Equation (10). These results demonstrate the importance of striking the optimal balance between the positive and negative effects of message bundling, which is further discussed with the results of the number of transmissions in the following section.

### 4.3. Number of Transmissions and End-to-End Delay

Unlike the ILP model of [17], the effect of packet retransmissions due to packet errors—especially on a link with a lower SNR—is taken into account in the objective function of the proposed INLP model through Equations (2)–(5). This effect of packet retransmissions is also properly captured in the Cooja emulation thanks to the Contiki-NG CSMA MAC protocol. Table 8 summarizes the average number of transmissions (including the original transmission) at the MAC layer.

**Figure 6:**
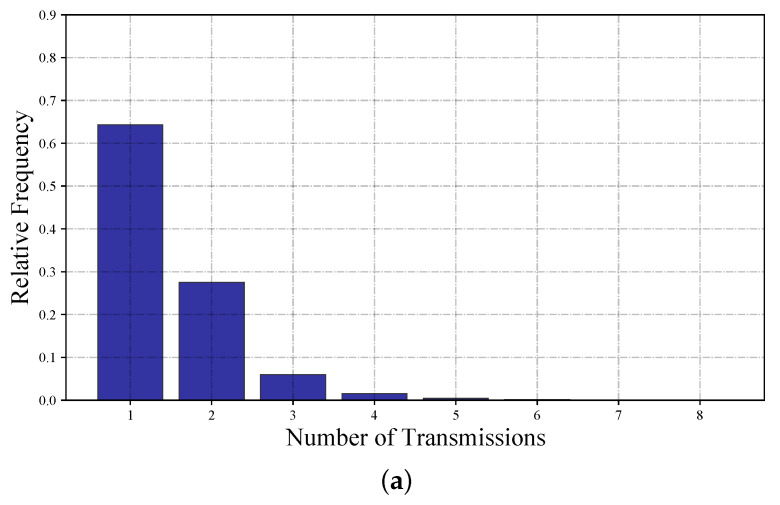

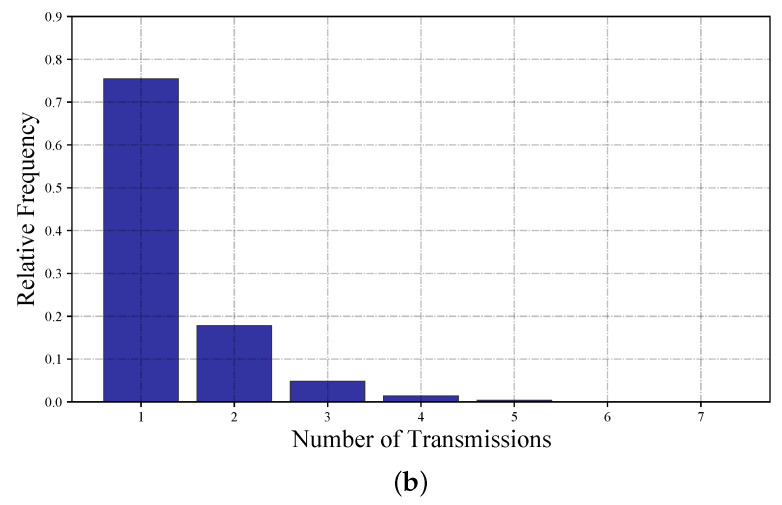
Distribution of the number of transmissions for T8: (**a**) ILP [17] and (**b**) INLP.

Except for the simplest topology of T2, INLP results in a lower number of transmissions than that with ILP. The difference between the two bundling schemes increases as the number of nodes increases; the difference in the average number of transmissions is 0.0906 for T8 compared to 0.0074 for T4. This difference in the number of transmissions is better illustrated through its distribution for the most complicated topology of T8, as shown in Figure 6.

Note that the mote output module of the Cooja emulator can display the E2E delay of each node. We observe that the requirement of user-defined synchronization accuracy, which is translated into the E2E delay of 6 seconds, is met for all of the cases considered, as shown in Table 9.

### 4.4. Discussion

The results of energy and power consumption based on Cooja emulation and Equation (10), respectively, clearly show the importance of taking into account the negative effects of message bundling—including increased PER and the number of retransmissions—as well as its positive effects. Thanks to the objective function that considers both the positive and negative effects of message bundling on the energy consumption, the proposed energy-efficient optimal bundling scheme can save more energy than the bundling based on ILP, which focuses only on the positive effects of message bundling.

The same results of energy and power consumption also show that Equation (10), based on the empirical model of the amount of energy required for the transmission of one information bit based on PER [6], captures the effect of packet retransmissions due to packet errors well; as discussed in Section 4.2, the results based on the Cooja emulation employing the CSMA MAC protocol match well with those based on Equation (10).

Note that, though the energy consumption measurement with the Cooja emulation has many advantages over that with actual hardware, as mentioned in Section 1, the results of energy consumption presented in Section 4.2 should be interpreted appropriately; the numerical results based on Equation (10) verify those based on the Cooja emulation by providing similar trends, but they cannot completely guarantee the accuracy of the energy consumption measurement with the Cooja emulation in comparison with that with actual hardware. In this regard, the energy consumption results presented in this section are to be interpreted as the relative performance of the bundling schemes, but not the absolute performance.

## 5. Conclusions

In this paper, we proposed a new energy-efficient optimal message bundling scheme for WSNs. Unlike the ILP model of [17], where the energy consumption is indirectly reduced by maximizing the message bundling number, the proposed scheme directly minimizes the power consumption of sensor nodes by considering relayed traffic from descendant nodes, as well as self-generated traffic, under the same constraints of E2E delay and synchronization accuracy. We formulated the optimal message bundling problem based on the power consumption of the entire WSN as an INLP model, which can take into account both the positive and negative effects of message bundling on the energy consumption, including packet retransmissions, and used SGHS to find an optimal solution. The experimental results based on the Cooja emulator of Contiki-NG demonstrate that the proposed optimal bundling scheme saves up to 51.8% and 8.8% of the total energy consumption with respect to the baseline case of no message bundling and the state-of-the-art ILP model of [17], respectively.

As a future extension of the current work, it is worth investigating an alternative formulation of the energy-efficient optimal bundling problem that enables analytical approaches (e.g., convex optimization), possibly with the approximation of the nonlinear objective function, given the complexity of the INLP model based on the nonlinear objective function for sensor nodes’ energy consumption and the solution procedure based on SGHS.

## Figures and Tables

**Table 1:** Contiki-NG vs. TinyOS.

	Contiki-NG	TinyOS
Protocols	IEEE 802.15.4, 6LoWPAN ${}^{1}$, RPL ${}^{2}$, and CoAP ${}^{3}$	
Language	Generic C	Dedicated NesC and C
Compiler	C compilers	Dedicated compiler
Portability	Easy	Hard
Main target	Industrial applications	Teaching & research

^1^ IPv6 over Low-Power Wireless Personal Area Networks [31]. ^2^ Routing Protocol for Low-Power and Lossy Networks [32]. 3 Constrained Application Protocol [33].

**Table 2:** Experimental environment used with Cooja.

Component	Description
Contiki-NG	IoT OS
Energest	Contiki-NG’s energy monitor
PowerTracker	Cooja’s radio energy monitor
Zolertia Z1 platform	WSN mote

**Table 3:** Default parameter values for the WSN and optimal bundling.

Parameter	MI [s]	*L_H_* [byte]	*L_M_* [byte]	$D_{e2e}^{max}$ [s]	$D_{e2e}^{SA}$ [s]	$\chi_{min}$	$\chi_{max}$	SNR [dB]	*E_TX_* [mJ/byte]
Value	1	23 *	10 ^†^	6	6	1	11 ^‡^	6	0.000032 **

* Based on the IEEE 802.15.4 data frame, 21-byte MAC header, and 2-byte MAC footer [38]. ^†^ Based on a six-byte timestamp, three-byte measurement data, and one-byte node ID. ^‡^ Based on the maximum payload size of 127 bytes [38]. ** Based on Z1’s CC2420 power levels: 3, 7, 11, 15, 19, 23, 27, and 31. In the CC2420 datasheet, at power level 31, the TX power P_TX_ is 0 dBm (1 mW).

**Table 4:** Parameter settings of SGHS for optimal bundling.

Parameter	Value	Description
*fun*	*P^total^*	Objective function
***x***	Γ	Vector of bundling numbers
***A***	–	Constraints matrix (i.e., ***A*** · ***x*** ≤ *limit*) according to Equation (15)
*limit*	6	Delay constraint (i.e., $min\left(D_{e2e}^{max},D_{e2e}^{SA}\right)$)
lb, ub	1, 10	Lower/upper bounds (i.e., $\chi_{min}$, $\chi_{max}$)
NI	9000	The number of improvisations (iterations)
HMS	2000	Harmony memory size
$HMCR_{m}$	0.2	Average harmony memory considering rate
$PAR_{m}$	0.2	Average pitch adjusting rate
$BW_{Max}$	6	Bandwidth upper bound
$BW_{Min}$	1	Bandwidth lower bound

**Table 5:** Optimal bundling numbers.

Topology	INLP (Equation (17))	ILP [17]
T1	6, 6	6, 6
T2	4, 4	6, 3
T3	6, 4, 4	6, 4, 4
T4	6, 4, 2	6, 6, 1
T5	6, 4, 4, 4	6, 4, 4, 4
T6	4, 4, 3, 2	6, 6, 3, 1
T7	5, 5, 5, 5, 5	5, 5, 5, 5, 5
T8	5, 6, 3, 3, 1	5, 6, 6, 1, 1

**Table 6:** Current consumption at different states of the Z1 mote [35].

State	Off	Down	Idle	Radio RX	Radio TX
Current	<1 μA	20 μA	426 μA	18.8 mA	17.4 mA

**Table 7:** Summary of the energy saved by the proposed optimal message bundling scheme (INLP) with respect to the baseline (no bundling) and the ILP model [17].

Topology	w.r.t. Baseline (No Bundling)		w.r.t. ILP [17]	
	Saved Energy [mJ]	$\eta_{\mathrm{base}}$ [%]	Saved Energy [mJ]	$\eta_{\mathrm{ILP}}$ [%]
T2	0.9030	51.8342	0.0058	0.6862
T4	2.1152	50.5915	0.1976	8.7295
T6	3.9040	51.0843	0.1463	3.7663
T8	6.0804	50.2176	0.5811	8.7922

**Table 8:** Average number of transmissions at the CSMA MAC layer.

Topology	Bundling Scheme	
	ILP [17]	INLP
T2	1.3007	1.3333
T4	1.2911	1.2837
T6	1.3080	1.2624
T8	1.4684	1.3778

**Table 9:** End-to-end delay performance.

Topology	Bundling Scheme	E2E [ms]		
		Max.	Min.	Avg.
T2	ILP	2312	64	1122
	INLP	3352	72	1650
T4	ILP	4352	88	2357
	INLP	1344	72	647
T6	ILP	4440	104	2375
	INLP	3368	88	2261
T8	ILP	5184	168	2519
	INLP	2520	152	1294

## Data Availability

Not applicable.
